# The Effects of Different Types of Eccentric Overload Training on Strength, Speed, Power and Change of Direction in Female Basketball Players

**DOI:** 10.3390/jfmk5030050

**Published:** 2020-07-16

**Authors:** Joey O Brien, Declan Browne, Des Earls

**Affiliations:** HealthCore, Department of Science and Health, Institute of Technology Carlow, Carlow R93 V960, Ireland; declan.browne@itcarlow.ie (D.B.); des.earls@itcarlow.ie (D.E.)

**Keywords:** flywheel, inertial, strength, speed, power, change of direction

## Abstract

The aim of this study was to investigate the effect of two types of eccentric (ECC) overload training on strength, speed, power and change of direction in female basketball players. Twenty amateur basketball players (mean ± SD: age: 23.67 ± 6.05 years; height: 1.73 ± 0.05 m; body mass: 80.28 ± 17.67 kg) participated in a randomized trial. The players performed either flywheel inertial training (FIT) (*n* = 11) or tempo ECC training (TET) (*n* = 9) for 4 weeks, performing two sessions weekly. Performance characteristics, one repetition back squat (1RM), counter-movement jump (CMJ), squat jump (SJ), 10-metre sprint (10 m), change of direction (COD) and sit and reach flexibility (S&R) were tested pre and post intervention. Post-hoc testing revealed significant improvements in the FIT group for 1RM (*p* ≤ 0.001; ES = 0.59), 10 m (*p* = 0.003; ES = −0.54) and CMJ (*p* ≤ 0.001; ES = 1.04), while significant improvements were revealed in the TET group for 1RM (*p* = 0.007; ES = 0.71) and S&R (*p* ≤ 0.001; ES = 0.58). In conclusion, both FIT and TET groups demonstrated a positive training stimulus for increasing muscular strength. FIT may produce superior adaptions in CMJ and 10-m sprint, while TET may produce superior adaptions in S&R. Neither group achieved increases in either SJ or COD.

## 1. Introduction

Basketball involves recurring bouts of high-intensity actions, such as sprinting, sudden stops, fast changes of direction, lateral shuffling, explosive jumping, and high force landings [1]. It is suggested that players require explosive strength and rate of force development of the lower body, agility with and without the ball, and speed over short distances. The available literature advises that strength [2], speed [3], power [4] and change of direction [5] are essential characteristics of basketball and that improving these will lead to increased game performance.

A past systematic review [6] suggests that muscular strength can enhance the ability to perform sport-specific tasks such as sprinting, jumping, and change of direction. Furthermore, similar research [7] indicated that stronger athletes produce superior performances during sport-specific tasks. Resistance training to improve muscular strength is commonly practiced in team sports, it customarily comprises of both ECC and concentric (CON) phases of movement. ECC muscle actions occur when the load applied to the muscle exceeds the force produced by the muscle itself, resulting in a lengthening action [8] with muscle forces tending to be highest during the ECC phase [6]. The CON strength of the athlete typically dictates the load, which leads to an inferior load during the ECC phase. Previous research [9] reported that resistance training programs that adequately load the ECC phase of a muscle contraction could elicit larger neuromuscular adaptations compared with traditional resistance training; highlighting this, Clark et al. [10] reported that a 4-week ECC overload training intervention produced favourable neuromuscular adaptations. Strength, power, and speed appear to be exceptionally responsive to ECC stimuli, and due to the potential adaptations with ECC training, it has become an increasingly popular component of strength and conditioning programs.

There are many types of ECC training with tempo ECC training (TET), and flywheel inertial training (FIT) implemented commonly in applied contexts. TET involves altering the time parameters placed on the ECC phase of an exercise [11] in an attempt to elicit enhanced ECC stimuli by overloading the ECC muscle action. Due to the greater force-producing capacity of ECC muscle, TET may not overload the ECC muscle action with regards to intensity but instead increase the duration of muscle tension throughout the ECC phase of an exercise [12]. This increase in muscle time under tension has been implemented in earlier work [13] to improve muscular strength. A 4-s TET showed larger increases in muscular strength when compared to a 1-s TET protocol, prompting authors [13] to conclude that slower muscular ECC actions are more effective in inducing muscular strength. Similar research [14] showed that adding 2 s to the ECC action in matched training protocols increases muscle activation. Contrary to these findings, Rea et al. [15] reported a significant increase in 1RM squat during a 2-s ECC phase when compared to 4 s, with no difference recorded in counter-movement jump height between both protocols. Supporting this, previous work [16] reported no significant difference in muscular strength between 2-s and 4-s ECC protocols; it should be noted, however, that a significant increase in flexibility was found in the slower ECC group. Results are conflicting, and further research on TET is warranted.

FIT was first introduced as a device for space travellers exposed to non-gravity environments [17]. The last decade has witnessed a growth in the practice of FIT with both amateur and semi-professional athletes [18,19] as a means to improve athletic performance. When using FIT devices, the rotation of the device’s flywheel commences with an ECC muscle action winding the flywheel’s strap. This is followed by a CON muscle action unwinding the flywheel’s strap, which immediately produces subsequent CON-ECC cycles. The force applied in the ECC action to bring the flywheel to a stop will rely on the kinetic energy generated during the CON action and also the strategy to apply force to the last third of the ECC action [20]. In simpler terms, the rate at which the strap is re-wound is based on the rate at which it is unwound [21], and if sufficient force is applied during the CON phase and effectively resisted in the ECC phase, an ECC overload can be achieved. A significant increase in strength and speed was reported in male soccer players after a 10 week FIT ECC overload intervention [18], subjects performed two weekly sessions using a flywheel device aimed at specific eccentric overloading of the hamstrings. This corresponds with the findings of Maroto-Izquierdo et al. [19], who reported positive effects in strength, speed, and power in male handball players after 6 weeks of FIT ECC overload training, the intervention group carried out 15 sessions of FIT in the leg-press exercise. Another study [22] reported improvements in sprint and power in youth soccer players after 10 weeks of in-season ECC overload training using FIT; subjects performed two FIT sessions weekly using the half squat and leg curl exercises. Previous work has focused on male athletes, and to the best of our knowledge, no study has investigated FIT effects on performance in female basketball players. This current study aimed to investigate the effect of two types of ECC overload training on strength, speed, power and change of direction in amateur female basketball players.

## 2. Materials and Methods

This research used a randomized trial to determine the effect of two types of ECC overload training on basketball specific performance characteristics. All the athletes were previously familiarized with the testing procedures. The tests included a maximal lower body strength test, lower body flexibility, short sprint, change of direction, and vertical jumps. The players were asked not to perform any strenuous exercise the day before each test, to consume their last meal at least 3 h before the scheduled tests, and to avoid caffeine supplementation for at least 24 h before the tests. Before the maximal trials, each subject performed a warm-up at 75% of their perceived maximum.

### 2.1. Subjects

Twenty amateur female basketball players were recruited for the study. The physical characteristics of the subjects are presented in Table 1. All subjects had a minimum of two years’ resistance training experience, although none of them had experience in FIT. The subject’s weekly training schedule consisted of three basketball training sessions and two strength/power sessions. The intervention took place during pre-season. Subjects were medically screened and excluded if any musco-skeletal injuries had occurred six months before the intervention. Before giving their written informed consent to participate, athletes were informed of the purposes, benefits, and risks involved in the study while also being made aware that they could terminate their participation in the study at any time. The study was cleared for ethical consideration from the Institute of Technology Carlow ethics committee before commencing; study number C00232530, approved on 28 May 2019.

### 2.2. Testing

Testing commenced with a dynamic warm-up that lasted approx. 15 min. The warm-up consisted of 5 min of low-intensity jogging, preceded by 3 min dynamic stretches, targeting the gluteal, hamstrings, adductors, quadriceps, and gastrocnemius, and 5 min of bodyweight exercises such as inchworms, press ups, squats, and plank holds.

### 2.3. Maximal Dynamic Strength Assessment

Maximal dynamic strength was assessed using a 1RM back squat to a depth of 90° knee flexion. Subjects were instructed to stand feet shoulder-width apart with an unloaded Olympic bar across the top of the shoulders and instructed to perform 5 repetitions. They were informed when they reached 90° knee flexion and then instructed to return to full knee extension (180°). The warm-up consisted of 5 repetitions at 40–60% of their perceived 1RM; after 3 min of full recovery, they performed 3 repetitions at 60–80% of their perceived maximum. The load was continuously increased until athletes could not perform another trial with the required technique. To consider a repetition valid, a full knee extension (180°) had to be reached, starting from a 90° knee flexion, 1RM value was obtained using as few attempts as possible.

### 2.4. Vertical Jump

Counter-movement jump (CMJ) and squat jump (SJ) heights were assessed using a contact platform (Chrono Jump, Bosco System, Barcelona, Spain) and the associated software (ChronoJump1.5.0). Subjects were instructed to keep their hands on their hips for the duration of all jumps. Three attempts were given for each jump, with subjects being instructed to jump as high as possible with the best jump of the three recorded for analysis. The CMJ started from a standing position without any pause between the ECC and CON phase; knee flexion was self-selected by the subjects. The ECC phase was eliminated from the SJ with subjects starting from 90° knee flexion. A 60 s recovery was given between each jump.

### 2.5. Speed

Sprint speed was evaluated by 10 m sprint times from a standing start. The front foot was placed 0.5-m before the first timing gate. Subjects were instructed to drive off from the starting line and sprint through all sets of timing gates. Timing gates (Witty Timing System, Microgate, Bolzano, Italy) were placed at 0- and 10-m meter marks. Subjects performed three sprints with 3 min recovery between each attempt. The fasted time was recorded for further analysis.

### 2.6. Change of Direction

Subjects completed the 505 test to assess change of direction performance, using the same standing start as per the sprint tests. Timing gates (Witty Timing System, Microgate, Bolzano, Italy) were set up at 10 and 15 m from the start line, respectively. Subjects ran 15 m, crossing through the timing gates at 10 m, made a 180° turn at 15 m, and ran 5-m back through the timing gates. Subjects were instructed to place either the left or right foot, depending on the trial, on or behind the turning line, before sprinting back through the gate. Three attempts on each side were given with 3 min recovery between attempts. If subjects changed direction before the designated line or turned off the incorrect foot, then the trial was disregarded and re-attempted when adequate recovery was provided [23]. The fasted attempt on each side was recorded, with the average used for analysis.

### 2.7. Flexibility

Flexibility was assessed using the sit and reach test, which has been used previously to assess lower back and hamstring flexibility [16]. Subjects were instructed to remove their shoes and placed their heels against the back of the flexibility box with legs stretched out straight. They then reached forward with arms and hands stretched out as far as possible, holding that position for 3 s. Three efforts were performed with a 1 min recovery given between each effort. The best attempt was recorded in meters for data analysis.

### 2.8. Training Intervention

Subjects were randomly assigned into two groups, TET (*n* = 9) and FIT (*n* = 11). No control group was used as it would only show that both treatments had an effect against it, effect size was instead utilized to suggest if one training group was superior to the other. Both training groups participated in 4 weeks of supervised training with two training days per week. A recovery period of 48 h was given between sessions to allow for adequate recovery. The TET group performed four sets of eight repetitions at 65% 1RM barbell back squat using 2-s CON and 4-s ECC phases, and two-minute intra-set rest periods were given. FIT training sessions consisted of four sets of ten repetitions on a flywheel device (kBox 3, Exxentric, AB TM, Bromma, Sweden) with two-minute intra-set rest periods given. Both the first and second repetitions of each set were used to “increase momentum” [21] and were not classed as working sets. An inertial load of 0.075 kg m² was used in the FIT group, which is the recommended inertial loading to maximize ECC overload [24]. Two familiarisation sessions were attended by the FIT group, allowing subjects to familiarise themselves with the device and exercise. Foot placement was standardized, with subjects standing directly over the drive belt. Subjects were instructed to perform the CON phase as fast as possible, then resist the inertia force gently during the first third of the ECC action, and then apply maximal effort to stop the movement at the end of the range of motion [12]. This training session schedule is similar to others used in the literature with flywheel devices. Both groups performed the same total amount of repetitions weekly.

### 2.9. Statistical Analysis

Between-group baseline differences in anthropometric characteristics were verified using the independent sample *t*-test. Data are presented as means and standard deviations (SD). The normality of data was tested using the Shapiro–Wilk test for all variables. To establish the effect of the interventions on the dependent variables, a 2 (group: FIT and TET) × 2 (time: pre, post) ANOVA with repeated measures was determined for each parameter. When group × time interactions were statistically significant, group-specific Bonferroni post hoc tests were applied. Effect sizes were calculated using Hedge’s g and can be interpreted as <0.2, 0.2–0.49, 0.5–0.79, >0.8 to represent small, trivial, moderate and large effects, respectively [25]. The level of significance was set at *p* < 0.05.

## 3. Results

**Figure 1:**
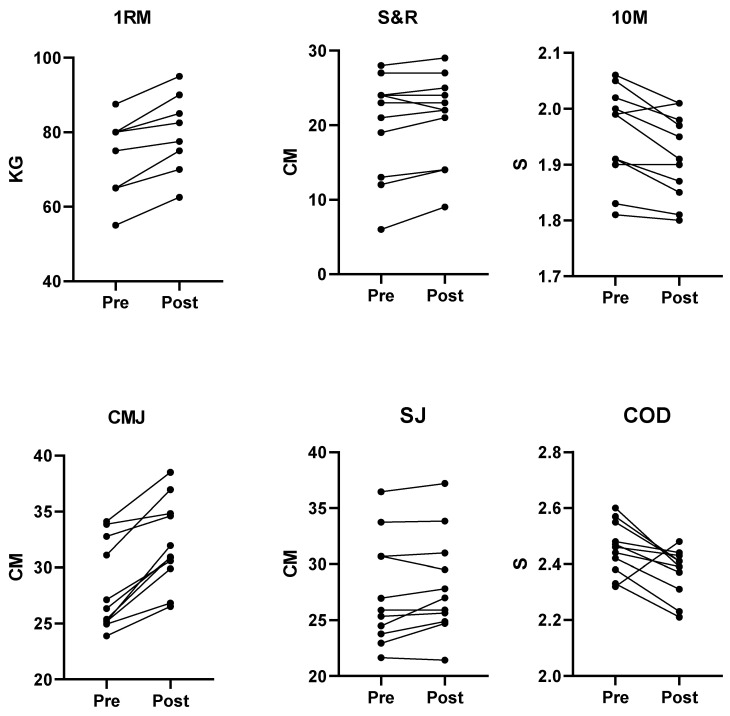
Pre and post intervention data for FIT group. Abbreviations: 1RM; 1 rep maximum squat test, S&R; sit and reach test, 10 M; 10 metre sprint test, CMJ; counter-movement jump, SJ; squat jump, COD; 505 change of direction test.

**Figure 2:**
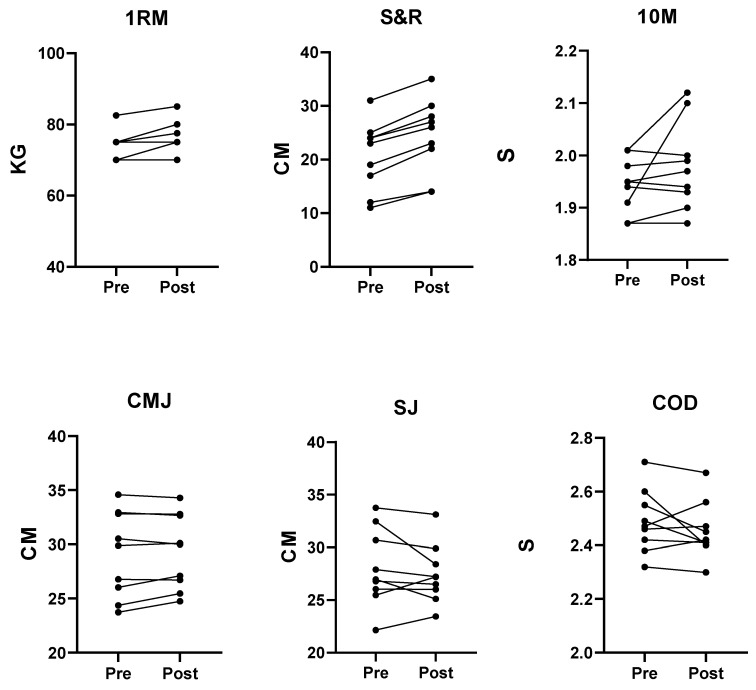
Pre- and post-intervention data for TET group. Abbreviations: 1RM; 1 rep maximum squat test, S&R; sit and reach test, 10 M; 10 metre sprint test, CMJ; counter-movement jump, SJ; squat jump, COD; 505 change of direction test.

Subjects from both groups completed all required training sessions. Table 1 displays anthropometric characteristics for individual groups. Table 2 displays 1RM, S&R, 10 m, CMJ, SJ, and COD (Mean ± SD) for both training groups. Individual subject data from both groups is displayed in Figure 1 and Figure 2. There was a main effect of time for 1RM (F = 64.4, *p* ≤ 0.001, η_p_^2^ = 0.07), S&R (F = 69.9, *p* ≤ 0.001, η_p_^2^ = 0.79), 10 m (F = 0.001, *p* = 0.97, η_p_^2^ = 0.01), CMJ (F = 49.9, *p* ≤ 0.001, η_p_^2^ = 0.74), COD (F = 8.42, *p* = 0.01, η_p_^2^ = 0.32). There was a significant variable by group interaction for 1RM (F = 9.12, *p* = 0.007, η_p_^2^ = 0.01), S&R (F = 28.2, *p* ≤ 0.001, η_p_^2^ = 0.61), 10 m (F = 10.14, *p* = 0.005, η_p_^2^ = 0.36), CMJ (F = 19.21, *p* ≤ 0.001, η_p_^2^ = 0.52). Post hoc analysis showed a 1RM performance increase for both FIT (*p* ≤ 0.001; ES = 0.59, moderate increase) and TET (*p* = 0.007; ES = 0.71, moderate increase) groups, a S&R performance increase for the TET group (*p* ≤ 0.001; ES = 0.58, moderate increase), a 10 m performance increase for the FIT (*p* = 0.003; ES = −0.54, moderate decrease) group and a CMJ performance increase for the FIT (*p* ≤ 0.001; ES = 1.04, large increase) group.

## 4. Discussion

The present study examined the effect of two types of ECC overload training on basketball-specific performance characteristics. To the authors’ knowledge, it was the first study to examine ECC overload training modalities in female amateur basketball athletes. The main findings of the present study were that FIT was successful in improving strength, speed, and CMJ, while TET was successful in improving strength and flexibility.

Our study found an increase in 1RM back squat (*p* < 0.001; ES = 0.59), which coincides with earlier research [19] showing that FIT can increase maximum lower body strength. The subjects (*n* = 15) performed 15 sessions of FIT over 6 weeks. Authors reported a 12.2% increase in 1RM leg press in male handball players, with authors attributing the increase to improved neural adaptions derived from the ECC overload provided by FIT. Strength increases have been discussed as originating from neural factors such as increased neural drive, altered motor unit firing rate, enhanced motor unit synchronization and this may be magnified by the ECC overload derived from FIT, with increased motor unit recruitment required for breaking the flywheel inertia during the return movement [21].

There was also a statistically significant increase in the TET group in 1RM back squat, although less than the FIT group. Previous work [26] reported a 30% increase in maximum lower body strength (leg press exercise) in 14 women over a 10-week training period, using the same 4-s ECC/2 s CON protocol as our study. It should be noted that the subjects were described as sedentary with no resistance training experience. Similar findings have been reported by Kim et al. [16], reporting a 14.9% increase in 1RM after a 4-week intervention. Subjects performed only one set until momentary muscular fatigue with 10-s CON and ECC phases at 50% of 1RM; subjects from this study were described as physically active female college students. Both studies show greater ∆% increases than our study, but this may be due to the methodological differences, and also the relatively low training age of subjects when compared to our study. A past investigation [27] reported that traditional resistance training produced superior peak and mean propulsive forces compared to TET; however, the time under tension was higher in the TET group. This observation may explain as to why the TET group may increase muscular strength (more time under tension). Both FIT and TET training protocols may be appropriate training modalities to improve maximal lower body strength, although FIT may yield greater results. The improvements in strength during this training intervention are most likely because of improved neural activation of muscle rather than muscle hypertrophy.

A positive effect on speed (*p* ≤ 0.03; ES = 0.54) was also established during the current study in the FIT group. The FIT group decreased the 10-m sprint time by 2%, which coincides with De Hoyo et al. [22], who found a small increase in the 10-m sprint performance (ES = 0.15) in 18 elite junior soccer players. Subjects performed a weekly FIT session (both bi-lateral flywheel squat and flywheel leg curl) over 10 weeks. The improvement in acceleration tasks was attributed to improvements in quad muscular strength. Multi-joint lower body strength training is considered relevant to improve sprinting as sprinting requires powerful extensions of the hip, knee, and ankle joints [28]. A similar study [18] on male footballers recorded a large improvement (ES = 0.80) in a 30-m sprint after a FIT (flywheel leg curl) training intervention, which is a larger effect than found in our study (ES = 0.54) and much larger than the De Hoyo et al. [22] study (ES = 0.15). It may be possible that the hamstrings are more involved during longer when compared to shorter, linear-sprinting distances. A hamstring-dominant exercise (flywheel leg curl) was incorporated by Askling et al. [18], which may explain the in-study differences. A similar study [29] investigated the effect of adding a weekly ECC-overload training session (bi-lateral flywheel squat) on strength and athletic performance in male handball players. They reported a 2.3% performance increase in their 20-m sprint performance during a 7-week training program, which coincides with our study. Independent of the exercises used, the enhanced ECC overload reached by FIT may be an effective stimulus when trying to improve sprint performance.

There was a large increase (∆ 10%; *p* ≤ 0.001; ES = 1.04) in CMJ performance in the FIT group. These findings are similar to those of [22,29] who both reported significant increases in CMJ performance, ∆ 6.2%, ES = 0.57 and ∆ 7.3%, ES = 0.58 respectively, supporting FIT as a training paradigm when aiming to increasing CMJ performance. In agreement with this, comparable research [30] compared the effects of two different inertial loads (one group used the mass of the flywheel the other group used 10 kg heavier) on lower body power in 58 male physical education students, with both groups seeing significant improvements, ∆ 3.8 and 6.7% respectively. ECC overload training appears to be effective in promoting increases in capacities highly related to athletic performance, such as vertical jump. The efficacy of FIT to promote functional and structural adaptations is possibly mediated by the capacity to achieve higher forces during the ECC muscle action, which maximizes the stretch–shortening cycle and, thus, the capacity to produce greater force in the subsequent CON action. It has been suggested [19] that flywheel RE training could emphasize the stretch reflex and the stretch–shortening cycle use, which would boost neural adaptations after a period of training. No significant improvement was recorded for the TET group in CMJ performance, which may be due to the slower ECC phase during TET, which may not enhance the stretch–shortening cycle use. 

Concerning SJ performance, there was no significant increase found in either the FIT or TET group. Our findings contradict those of Naczk et al. [30] who reported significant increases in SJ performance in two groups using two different inertial loads (ES = 0.65 & ES = 0.91), and Maroto-Izquierdo et al. [19] who reported a moderate increase (ES = 0.54) in male handball players after FIT training programs. Moreover, a prior investigation [31] was again in contradiction of our findings, reporting a performance increase in SJ after 6 weeks of FIT in both males and females (∆ 4 & 8% respectively). It should be noted however, that although the FIT training group did not record statistical significance in our study, they did show a small performance increase (∆ 2.1%).

The current study also established no statistical significant increase in COD performance in either the FIT or TET group, while performance improvements were recorded, ∆ 3.7% and 1.4%, respectively. COD requires complex skills, and factors other than strength may have a more considerable impact. Specificity, which refers to the degree of similarity between training exercises and athletic performance, is said to be an essential factor for strength transfer to exercise performance [32]. COD may need a more specific training paradigm to afford such a transfer. Regarding flexibility, a 17.6% performance improvement was observed in the TET group, which corresponds with previous work [16], who reported a 14.7% increase using the same 4 s ECC/2 s CON TET protocol. Slower ECC movements may cause the muscles to be in a lengthened position for a more extended period, which may provide an added stimulus to improve flexibility. No significant increase in flexibility was recorded in the FIT group.

In conclusion, both FIT and TET groups demonstrated a positive training stimulus for increasing muscular strength. FIT may produce superior adaptions in CMJ and a 10-m sprint, whereas TET may produce superior adaptions in flexibility. Neither group achieved increases in either SJ or COD. The results of the present investigation have practical applications when recommending ECC training to female basketball players aiming to improve strength, speed, flexibility and vertical jump height. Our study suggests that two weekly FIT sessions may significantly improve 1RM parallel back squat, 10-m sprint and CMJ performance over 4 weeks, while two weekly TET sessions may improve 1RM parallel back squat and sit and reach performances, additionally FIT or TET does not appear to improve COD or SJ performance.

## Figures and Tables

**Table 1:** Descriptive data of the participants, Mean ± SD.

	Age (y)	Height (cm)	Weight (kg)
FIT Group	23.17 ± 5.55	171.41 ± 6.37	77.93 ± 19.05
TET Group	24.18 ± 6.56	174.53 ± 4.32	82.64 ± 16.30

**Table 2:** 1RM, S&R, 10 m, CMJ, SJ and COD results for pre and post intervention in both FIT and TET intervention groups. Values displayed as Mean ± SD.

	FIT			TET		
	Pre	Post	% Change	Pre	Post	% Change
1RM (kg)	71.59 ± 10.57	77.73 ± 10.57 **	8.6	73.61 ± 3.93	76.39 ± 3.93 *	3.8
S&R (cm)	20.09 ± 6.61	20.91 ± 5.82	0.1	20.67 ± 6.13	24.3 ± 6.13 **	17.6
10 m (s)	1.95 ± 0.08	1.91 ± 0.07 *	−2	1.94 ± 0.05	1.98 ± 0.08	1.9
CMJ (cm)	28.17 ± 3.77	31.05 ± 3.66 **	10.2	29.08 ± 3.76	29.32 ± 3.28	0.8
SJ (cm)	27.51 ± 4.52	28.08 ± 4.31	2.1	28.08 ± 3.45	27.43 ± 2.65	−2.1
COD (s)	2.46 ± 0.09	2.37 ± 0.08	−3.7	2.49 ± 0.11	2.45 ± 0.10	−1.4

Abbreviations: 1RM; 1 rep maximum squat test, S&R; sit and reach test, 10 M; 10-metre sprint test, CMJ; counter-movement jump, SJ; squat jump, COD; 505 change of direction test. * Significantly different from pre-training value, where * *p* < 0.01, and ** *p* < 0.001.
